# Gut bacteria reflect the adaptation of *Diestrammena japanica* (Orthoptera: Rhaphidophoridae) to the cave

**DOI:** 10.3389/fmicb.2022.1016608

**Published:** 2022-12-21

**Authors:** Yiyi Dong, Qianquan Chen, Zheng Fang, Qingshan Wu, Lan Xiang, Xiaojuan Niu, Qiuping Liu, Leitao Tan, Qingbei Weng

**Affiliations:** ^1^School of Life Sciences, Guizhou Normal University, Guiyang, Guizhou, China; ^2^Qiannan Normal University for Nationalities, Duyun, Guizhou, China

**Keywords:** insect, gut microbiota, adaptation, cave, 16S rRNA gene sequencing, light strength, amino acid metabolism

## Abstract

The gut microbiota is essential for the nutrition, growth, and adaptation of the host. *Diestrammena japanica*, a scavenger that provides energy to the cave ecosystem, is a keystone species in the karst cave in China. It inhabits every region of the cave, regardless of the amount of light. However, its morphology is dependent on the intensity of light. Whether the gut bacteria reflect its adaptation to the cave environment remains unknown. In this research, *D. japanica* was collected from the light region, weak light region, and dark region of three karst caves. The gut bacterial features of these individuals, including composition, diversity, potential metabolism function, and the co-occurrence network of their gut microbiota, were investigated based on 16S rRNA gene deep sequencing assay. The residues of amino acids in the ingluvies were also evaluated. In addition, we explored the contribution of gut bacteria to the cave adaptation of *D. japanica* from three various light zones. Findings showed that gut bacteria were made up of 245 operational taxonomic units (OTUs) from nine phyla, with Firmicutes being the most common phylum. Although the composition and diversity of the gut bacterial community of *D. japanica* were not significantly different among the three light regions, bacterial groups may serve different functions for *D. japanica* in differing light strengths. *D. japanica* has a lower rate of metabolism in cave habitats than in light regions. We infer that the majority of gut bacteria are likely engaged in nutrition and supplied *D. japanica* with essential amino acids. In addition, gut bacteria may play a role in adapting *D. japanica’s* body size. Unveiling the features of the gut bacterial community of *D. japanica* would shed light on exploring the roles of gut bacteria in adapting hosts to karst cave environments.

## Introduction

Microorganisms are abundant in the guts of animals, and most of them are beneficial for their hosts’ health and various physiological processes (Douglas, 2011; Engel and Moran, 2013), including nutrition, development, reproduction, defense, and detoxification (Douglas, 2015). For example, they provide nutrients that hosts cannot synthesize, such as essential amino acids, B vitamins, and sterols (Douglas, 2009, 2015). In contrast to mammals, the diversity of gut microbiota in insects varies considerably among different taxa, possibly as the consequence of complex diets, variation in life span, and gut physiology (Douglas, 2011). *Apis mellifera*, for instance, harbored 14 species; however, *Reticulitermes speratus* harbored 254 operational taxonomic units (OTUs) (Colman et al., 2012).

Karst is a soluble rock that dissolves in water to form stone forests on the ground and caves under the earth. It accounts for around 20% of the land on the earth that is not covered by ice and provides water to approximately 25% of the world’s population (Bystriakova et al., 2019). The karst area in southwestern China, the largest karst area in the world, is one of the 25 global biodiversity hotspots (Luo et al., 2016). The karst cave is characterized by darkness, moistness, constant temperature, poor air circulation, a high concentration of carbon dioxide, a lack of food, and low biomass (Lavoie et al., 2007). Multiple features of insect adaptations to cave environments include morphological adaptations (loss of vision and body color changes), physiological adaptations (low metabolic rate and CO_2_ tolerance), and behavioral adaptations (loss of circadian rhythm and variations in mating behavior) (Howarth and Moldovan, 2018). Therefore, cave inhabitants are an ideal model for exploring the environmental adaptations of organisms.

*Diestrammena japanica* Blatchley (Orthoptera: Rhaphidophoridae) is the dominant insect species in karst caves and is found in all regions of caves. However, individuals from different light strength exhibit distinct morphological features. Previous reports on *D. japanica* primarily focused on its population composition (Li et al., 2001; Li, 2006), the influence of environmental factors on its population structure (Li, 2006), and the heavy metal concentration in its body in caves with heavy metal pollution (Xu et al., 2010; Ye and Li, 2011). *D. japanica* has access to different foods depending on light strength. Specifically, species in the light region consume more moss and fern, while species in weak light and dark regions feed on more animal carcasses and fungi (Li, 2007). However, the gut bacterial compositions and their roles in the adaptation of *D. japanica* to cave habitats remain unknown.

In the study, 16S rRNA gene deep sequencing was utilized to unveil the composition and structure of gut microbiota in *D. japanica* near the entrance of karst caves (light region) and in cave habitats (weak light and dark regions). Results showed that Firmicutes were the most dominant phylum, and *Lactobacillus* was the most abundant genus. The community composition and diversity indices were not influenced by light strength. Phylogenetic investigation of communities by the reconstruction of unobserved states analysis implied that many gut bacteria were involved in nutrition, such as several amino acid biosynthesis pathways. Gut bacteria of *D. japanica* exhibit low diversity but strong cooperation interactions in the dark region. These results indicated that the gut bacteria may help *D. japanica* adapt to the poor nutrient cave environment. Exploring the diversity and function of gut bacteria from cave insects can help understand species adaptation in extreme environments.

## Materials and methods

### Sample collection

*Diestrammena japanica* was collected from three karst caves in Libo county, Guizhou province, China. The locations of the caves are shown in Supplementary Table 1. Based on the habitat categories, light strength in the cave environment, and the presence of *D. japanica*, the zone was divided into the light region (the areas with direct sunlight are near the cave’s entrance and distance from the entrance within 10 m), the weak light region (transition twilight areas distance from the entrance within 10–30 m), and the dark region (deep zones of the cave are entirely dark and distance from the entrance beyond 50 m), according to the light strength and distance from the cave entrance (Culver and Pipan, 2019). Species of *D. japanica* were caught and stored separately in sterile tubes at −20°C, and samples were subsequently transferred to the laboratory in 12 h. To quantify the individual size of *D. japanica*, a ruler with a 0.1-mm scale was used to measure trait features, including body length, hind leg length, front leg length, middle leg length, and head width, with 24 individual repetitions for each region. The least significant difference (LSD) test in *agricolae* package (de Mendiburu, 2014) was employed to examine whether there were significant differences in these morphological traits.

### Amino acid titer determination in ingluvies of *Diestrammena japanica*

Ten fresh species from each light region were dissected with dissection forceps. The inclusion from the ingluvies where food was stored was extracted in a 2-ml sterile vial and then stored at −80°C. An amino acid analyzer (Sykam S433, Fürstenfeldbruck, Germany) was used to assess the concentration of amino acids in foods.

### DNA extraction and 16S rRNA gene sequencing

The ethanol-treated samples were rinsed three times with enough sterile water in plates. The gut was then isolated from other tissues and placed in a sterile tube. A total of 15 guts from each light-strength region of the caves were pooled together as one biological replication. Guts were ground into a powder with a pestle. To avoid the contamination of microorganisms from the air in the laboratory, the above operations were carried out in a biosafety cabinet. DNA was extracted using the DNeasy PowerSoil Kit (Qiagen, Hilden, Germany) according to the manufacturer’s protocol. The DNA quality and quantity were measured with the BioTek Epoch 2 Microplate Spectrophotometer (Agilent, Palo Alto, USA). The bacterial V3–V4 hypervariable region of the 16S rRNA gene was amplified by the universal primers 338F (ACTCCTACGGGAGGCAGCAG) and 806R (GGACTACHVGGGTWTCTAAT) using TransStart FastPfu DNA polymerase (TransGen, Beijing, China) according to the manufacturer’s protocol. Each sample was amplified in triplicate, and then all polymerase chain reaction (PCR) products were checked by 2% agarose gel electrophoresis. High-quality PCR reaction products that were amplified from the same sample were pooled together and subjected to purification with AxyPrep DNA Gel Extraction Kit (Axygen Biosciences, Union City, CA, United States). Purified products were quantified with QuantiFluorTM-ST (Promega, United States) following the manufacturer’s protocol. The paired-end libraries were synthesized with the TruSeqTM DNA Sample Prep Kit (Illumina, San Diego, CA, United States) following the manufacturer’s protocol. Sequencing was conducted with Illumina MiSeq platform sequencing technology with a maximum read length of 2 × 300 bp at Shanghai Majorbio Bio-pharm Technology Co., Ltd.

### Analysis of 16S rRNA gene sequencing data

The quality of raw reads was assessed with FASTP (version 0.19.6) (Chen S. et al., 2018). High-quality reads were assembled with Flash (version 1.2.11) (Magoc and Salzberg, 2011) and analyzed with QIIME (version 1.9.1) and Mothur (version 1.30.2) (Schloss et al., 2009; Caporaso et al., 2010; Schloss, 2020). Furthermore, bacterial operational taxonomic units (OTUs) were grouped with 97% similarity to the SILVA (version 132) and Greengenes (version 135) database with the Ribosomal Database Project Classifier (version 2.11) (DeSantis et al., 2006; Edgar, 2013; Quast et al., 2013). OTUs with more than 20 reads for all samples and distribution across three samples (at least five reads for each sample) were retained for further analysis.

### Function prediction of gut bacterial communities

The function of gut bacteria was predicted by searching against the Clusters of Orthologous Groups (COGs) database with the Phylogenetic Investigation of Communities by Reconstruction of Unobserved States (PICRUSt) (Langille et al., 2013). These analyses were performed using the Majorbio Cloud Platform. ^1^ The Kruskal–Wallis test was used for multiple group comparisons. Differences were considered significant if the *p*-value was <0.05.

### Analysis of network for gut bacterial communities

To compare the structure and existence pattern of the gut bacterial communities of *D. japanica*, we performed the bacterial co-occurrence network for each light-strength region separately. Each co-occurrence network was conducted based on the top 50 relative abundance genera that occurred in eight phyla in the network analysis. Only the strong correlations in these groups were visualized in the co-occurrence network. Thus, the bacterial interactions comprised only significant correlations (*p* < 0.01) of the correlative coefficient (the absolute value of *r* > 0.8) in the network. Moreover, we calculated the edge, connectance, clustering coefficient, average degree, and modularity for each co-occurrence network. We also detected that the links per species in each network were significantly different (*p* < 0.001) in the random network using the r2dtable null model. All relative network analyses were performed in R software (R Development Core Team, 2018), using the packages *igraph* (Csardi and Nepusz, 2006), *bipartite* (Dormann et al., 2008), *psych* (Revelle and Revelle, 2015), and *statnet* (Handcock et al., 2008).

### Statistical analysis

First, the LSD test was conducted to assess whether there were significant differences in the morphological traits of *D. japanica* among the three light-strength regions. Mothur and R software were used to build rarefaction curves to determine if the sequencing depth (reads) was sufficient to capture the majority of gut bacteria (Schloss et al., 2009; Schloss, 2020). The top 35 abundance genera were used to generate a heatmap with ggplot2 (Ginestet, 2011), and the Kruskal–Wallis test was used to test the differences in the abundances of the 35 genera among three light regions. The Venn diagram, which exhibited the overlap of OTUs among groups, was generated with the R package *VennDiagram* (Chen and Boutros, 2011). To decipher whether the gut bacterial composition was affected by regions with different light-strength regions, the Shannon and Simpson diversity indexes were calculated with Mothur, and then the pairwise Wilcox test was used to test for significant differences in the bacterial diversity indices, with a *p*-value adjusted for false discovery rate (fdr-adjusted). Subsequently, the beta diversity of bacterial communities was compared *via* ANOSIM and permutational multivariate analysis of variance (PERMANOVA, permutation 9,999 times, based on the Bray–Curtis matrix), respectively. The analyses were conducted *via* MicrobiotaProcess (Xu et al., 2022) and Vegan (Oksanen et al., 2013) packages in R (version 4.1.2).^2^ A PERMANOVA test uncovered that the compositions of gut bacteria were not significant differences among the three light regions, and then we tested the differences between two groups (light region as one group, and weak and dark regions as another group); abund_jaccard matrix (considering both OTUs’ availability and abundance) was used for principal component analysis (PCA), combining with the Adonis analysis (permutation 9,999 times).

## Results

### Morphological adaptations of *Diestrammena japanica*

**FIGURE 1:**
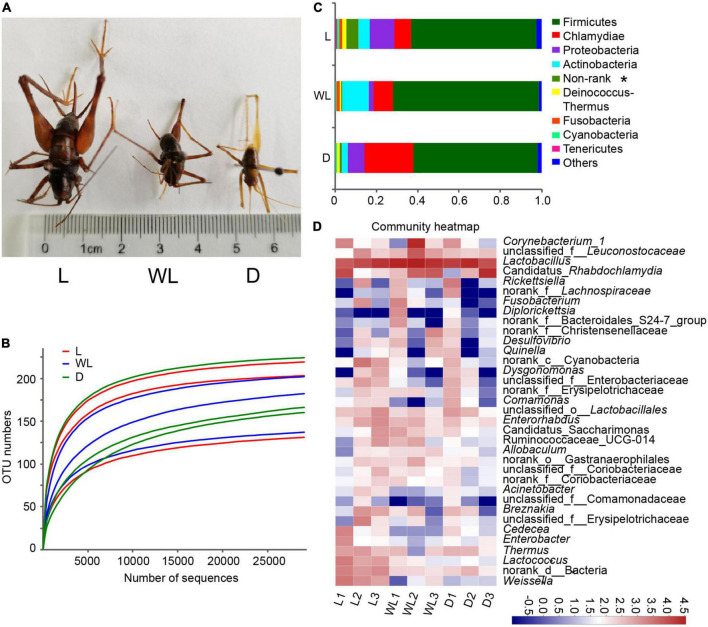
Gut bacteria dynamics among different light-strength regions in *Diestrammena japanica*. **(A)** Morphology of *D. japanica* from different light-strength regions. **(B)** Rarefaction curves generated from randomly subsampled data sets with the same number of 16S rRNA gene deep sequencing. **(C)** Relative abundance of gut bacteria at the phylum level. **(D)** Relative abundance of the top 35 abundance genera gut bacteria, the color scales display the bacterial relative abundance (log10 transformed). L, light region; WL, weak light region; D, dark region. These labels are the same as in the other figures.

*Diestrammena japanica* is distributed in all regions of caves, independent of light strength. However, the LSD test showed that species from light regions were significantly larger than those from weak light and dark regions (Figure 1A and Table 1). However, the species from weak and dark regions were not significantly different in body size (Table 1).

**TABLE 1:** The morphological traits of *Diestrammena japanica* in different light-strength regions (*N* = 24, Unit: mm).

Region	Body length	Hind leg length	Front leg length	Middle leg length	Head width
L	2.74 ± 0.09^a^	6.71 ± 0.18^a^	3.85 ± 0.11^a^	3.65 ± 0.08^a^	0.42 ± 0.02^a^
WL	1.90 ± 0.10^b^	5.00 ± 1.27^b^	2.98 ± 0.18^b^	2.84 ± 0.17^b^	0.27 ± 0.01^b^
D	1.89 ± 0.13^b^	4.84 ± 1.25^b^	2.94 ± 0.16^b^	2.69 ± 0.15^b^	0.30 ± 0.02^b^

Mean ± standard error is shown. L, light region of the cave; WL, weak light region of the cave; D, dark region of the cave. Different lowercase letters in the same column are significantly different based on the least significant difference test results.

### The sequencing overview of gut bacteria from *Diestrammena japanica*

Sequencing was performed on a total of nine samples from three distinct light-strength regions in three caves. Following filtering, the number of high-quality reads and bases was 388,019 and 172,857,092, respectively (Supplementary Table 2). The average read length ranged from 442 to 449 (Supplementary Table 2). The minimum number of reads per sample was 32,208. The rarefaction curve verified that the depth of sequencing was sufficient to capture the majority of bacterial species (Figure 1B). The reads were clustered into 245 OTUs, which shared >97% sequence identity (Supplementary Table 3). In addition to Firmicutes, Proteobacteria, Actinobacteria, Cyanobacteria, Tenericutes, Deinococcus-Thermus, Fusobacteria, Chlamydiae, and one non-rank phylum were detected in the gut of *D. japanica* (Figure 1C and Supplementary Table 3).

### Compositions and diversities of gut bacteria among different light-strength regions in *Diestrammena japanica*

Firmicutes were the most abundant in all samples, accounting for 60.87, 70.79, and 60.18% of bacterial groups of species from light, weak light, and dark regions, respectively (Figure 1C). Chlamydiae accounted for 8.05–23.77% of the bacterial composition from different light-strength regions. Proteobacteria accounted for 2.36–12.07% of the total bacterial sequences, of which *Rickettsiella*, including one unclassified OTU, was the most abundant genus of Proteobacteria. The top two genera of Actinobacteria were *Corynebacterium* and *Enterorhabdus*.

The top 35 abundance genera were used to generate the heatmap (Figure 1D). *Lactobacillus* was the most abundant genus, accounting for 37.35, 59.89, and 52.72% of bacteria found in species from light, weak light, and dark regions, respectively (Figure 1D and Supplementary Table 3). Among the 16 OTUs belonging to the genus *Lactobacillus*, *L. amylovorus* and *L. oligofermentans* were the most abundant, accounting for 66.38–85.46% and 2.43–10.41% of the abundance of the genus *Lactobacillus*. The abundance of Candidatus *Rhabdochlamydia* and *Corynebacterium* ranged from 8.05 to 23.77% and 1.43 to 11.20%, respectively.

Among the top 35 genera, *Lactococcus* (Kruskal–Wallis test, *df* = 2, *p* = 0.0376), *Weissella* (Kruskal–Wallis test, *df* = 2, *p* = 0.0358), and non-rank bacteria (Kruskal–Wallis test, *df* = 2, *p* = 0.0302) were more abundant in species from the light region than in those from weak light and dark regions (Figure 1D), and remailing bacterial groups were not significantly different among the three light regions.

**FIGURE 2:**
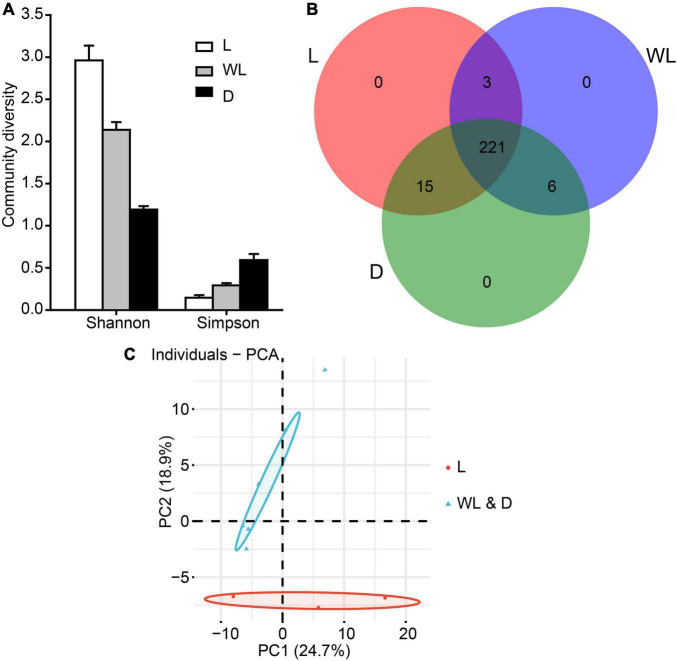
Gut bacterial community dynamics among different light-strength regions in *D. japanica*. **(A)** Community diversity measured by the Shannon index and Simpson index. **(B)** Overlap of OTUs from different light-strength regions. **(C)** Principal component analysis showed gut bacteria were clustered into two groups according to light strengths, based on the Adonis testing result (*R* = 0.3457, *p* = 0.049); the different colors show different groups with the boundaries of the 95% confidence ellipses. The red represents the species from weak light and dark regions.

Furthermore, the diversity analysis of bacterial community revealed that the Shannon index exhibited the following pattern: light (2.96) > weak light (2.14) > dark (1.19), and the Simpson index exhibited the opposite pattern to the Shannon index (Figure 2A). The two diversity indices were not significantly different among light-strength regions (all fdr-adjusted *p*-values from the pairwise Wilcox test were greater than 0.05, Supplementary Table 4).

### Composition similarity of gut bacteria among different light-strength regions in *Diestrammena japanica*

A total of 239, 230, and 242 OTUs, respectively, were detected in the species from light, weak light, and dark regions (Figure 2B). The Venn diagram analysis revealed that 221 OTUs were shared by species from three regions. species from the dark and weak light regions shared six OTUs, while species from the light region shared three and 15 OTUs with species from the weak light and dark regions, respectively (Figure 2B). At bacterial species levels, the samples from three light-strength regions revealed consistent patterns (no significant differences in bacterial composition) from ANOSIM (statistic *R* = 0.004, *p* = 0.43). However, the PERMANOVA test uncovered that the bacterial community did not have significant changes in three light-strength regions (Supplementary Table 5, PERMANOVA Pseudo-F = 1.39, *R*^2^ = 0.32, *p* = 0.13). Intriguingly, the PCA analysis with abund_jaccard matrix indicated that bacterial communities of species from weak light and dark regions were clustered as one group, and the remaining species from the light region were clustered as another group (Adonis test, *R* = 0.3457, *p* = 0.049; the first two axes explaining 43.60% of the total variability) (Figure 2C).

### Co-occurrence network analysis of gut bacteria among different light-strength regions in *Diestrammena japanica*

**FIGURE 3:**
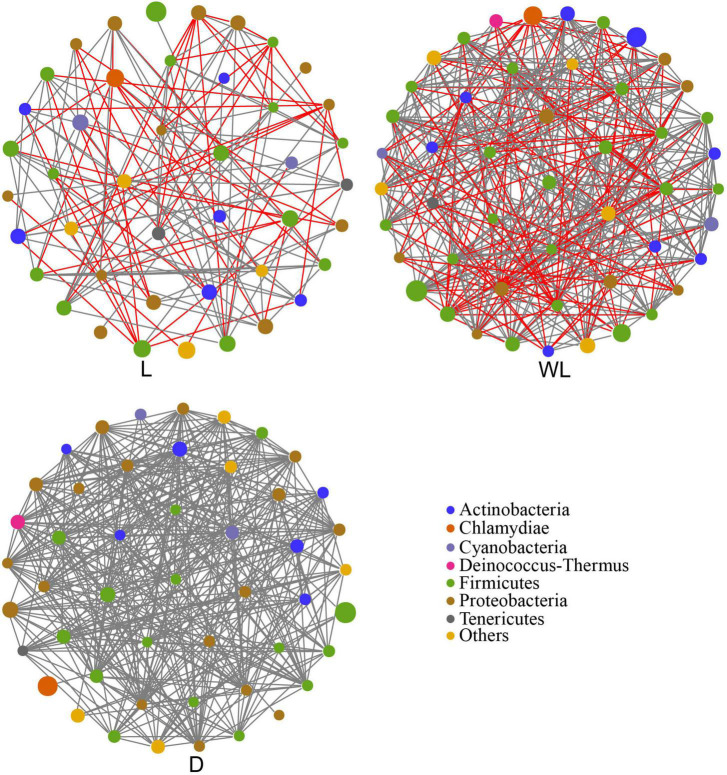
Co-occurrence network analysis of gut bacteria community in *D. japanica*. The size of the node represents the relative abundance. Positive interaction, gray line; negative interaction, red line.

A co-occurrence network analysis was carried out to unveil the interaction and structure of gut bacterial communities. Compared with gut bacteria in the species from the light region (edge: 138), the interactions among gut bacteria in the species from the weak light and dark region (edge: 363 and 385 separately) were more complex (Figure 3 and Table 2). Moreover, we observed that the network only included the positive interaction (gray line) in the dark region; the network from the weak light consisted of 233 positive edges and 130 negative edges (red line). However, there were 83 positive interactions and 55 negative interactions in the gut bacterial community from the light region. In addition, the connectance and average degree of the network highlighted the pattern of dark > weak light > light, while the modularity exhibited the reverse pattern (light > weak light > dark).

**TABLE 2:** The co-occurrence network parameter in different light-strength regions.

Region	Edge	Connectance	Clustering coefficient	Average degree	Modularity
L	138	0.15	1.00	6.41	0.75
WL	363	0.31	1.00	14.82	0.61
D	385	0.34	1.00	16.04	0.47

L, light region of the cave; WL, weak light region of the cave; D, dark region of the cave.

### Function prediction of gut bacteria among different light-strength regions in *Diestrammena japanica*

**FIGURE 4:**
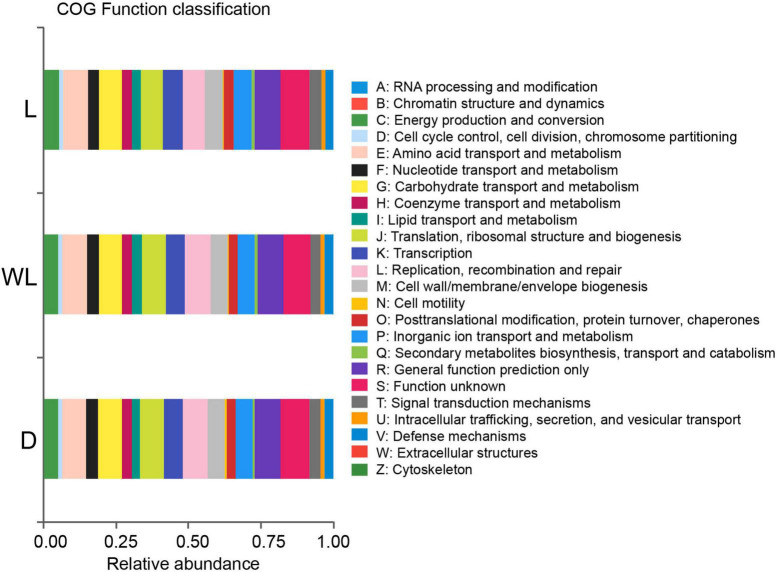
Relative abundance of gut bacterial function classification, inferred from PICRUSt analysis prediction.

The PICRUSt prediction was conducted to reveal the role of the gut bacterial community in *D. japanica*. The results uncovered that 34.96% of gut bacteria were involved in the nutritional function of the host. Specifically, 8.40, 8.21, 5.90, 5.26, 4.04, and 3.15% of bacteria, respectively, were involved in amino acid transport and metabolism, carbohydrate transport and metabolism, inorganic ion transport and metabolism, energy production and conversion, nucleotide transport and metabolism, and lipid transport and metabolism (Figure 4). Moreover, 2.52% of gut microorganisms were implicated in defense.

**FIGURE 5:**
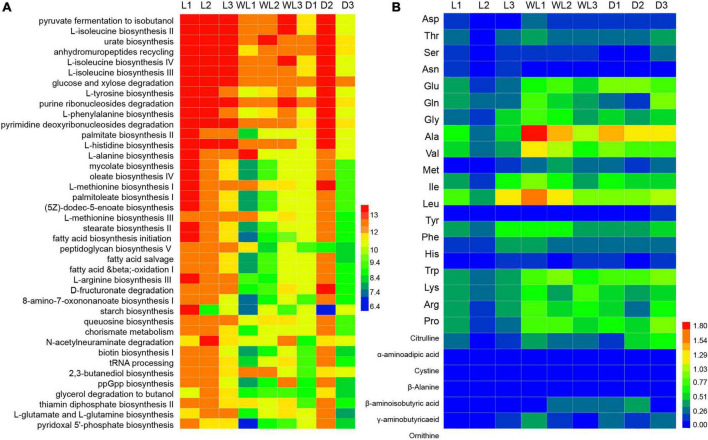
Abundance of metabolism pathway **(A)** and titer of amino acids **(B)**, the three letter codes represent the different amino acids, following the general amino acid code list, and the color scales indicate the abundances of metabolism pathways and amino acids.

At the same time, several pathways involved in methionine biosynthesis, including L-methionine biosynthesis I (Kruskal–Wallis test, *df* = 2, *p* = 0.0107) and L-methionine biosynthesis III (Kruskal–Wallis test, *df* = 2, *p* = 0.0107) were more abundant in species from the light region than in those from weak light and dark regions (Figure 5A). The titers of amino acid content in the food-storing ingluvies of *D. japanica* were further investigated. The titers of aspartic acid (Kruskal–Wallis test, *df* = 2, *p* = 0.0250) and methionine (Kruskal–Wallis test, *df* = 2, *p* = 0.0500) in the species from the light region were lower than in those from weak light and dark regions (Figure 5B). Moreover, the amino acid biosynthesis I (*r* = −0.8605, *p* = 0.0061) and III (*r* = −0.8388, *p* = 0.0092) pathways of gut bacteria were negatively correlated with the contents of methionine in food.

## Discussion

Diverse gut bacteria are potentially involved in many physiological processes of insects, which contribute to the adaptation of the host insects to the environment. In this study, the gut bacteria character of *D. japanica* in karst caves was determined by 16S rRNA gene deep sequencing. The compositions and diversity indices of gut bacteria of *D. japanica* did not exhibit convergent patterns influenced by light-strength regions in the caves. However, bacterial groups may provide diverse functions to *D. japanica* in regions of varying light strengths. Moreover, gut bacteria may have roles in the nutritional metabolism processes (amino acids and carbohydrates) and morphological adaptation of *D. japanica* in karst caves. Altogether, this study facilitates a step forward in understanding the roles of gut bacteria in the adaptation of insects in the nutrient-poor and dark cave environment.

### The overall feature of gut bacteria of *Diestrammena japanica*

In comparison to *Drosophila melanogaster* (139 OTUs), xylophagous long-horned beetles (average 103 OTUs), *Bombyx mori* (90 OTUs), and several wild mosquito species (5–71 OTUs) (Chandler et al., 2011; Engel et al., 2012; Chen B. et al., 2018; Zhao et al., 2019), *D. japanica* harbors more diverse gut bacterial composition. Each *D. japanica* sample from different light-strength regions was detected with more than 200 OTUs, covering eight bacterial phyla.

Host insects’ diets can affect gut bacteria composition (Colman et al., 2012; Paoletti et al., 2013; Yun et al., 2014). As a type of omnivorous insect with complex food structures, *D. japanica* has access to different food structures depending on the different light-strength regions. For example, species in the light region consume more moss and fern, whereas those in weak light and dark regions feed on animal carcasses and fungi (Li, 2007). Previous reports have shown a positive correlation between the complexity of diet structures and the diversity of gut bacteria (Zheng et al., 2021). Therefore, it is not unexpected that more numerous bacterial taxa were observed in the gut habitat of *D. japanica* than other insect taxa previously mentioned since they contribute to the decomposition of diverse food resources.

Generally, Firmicutes and Proteobacteria are the prominent groups of gut bacteria in insects (Yun et al., 2014; Chen et al., 2016; Vilanova et al., 2016; van Schooten et al., 2018). Similar to *D. japanica* of this study, the gut bacterial communities of *Epicauta longicollis*, *Megetra cancellata*, and *Bactrocera dorsalis* were dominated by Firmicutes (Colman et al., 2012; Andongma et al., 2015). However, in the intestinal habitat of some species, such as *Bombyx mori* and *Chrysoperla sinica*, Proteobacteria was the dominating phylum (Chen B. et al., 2018; Zhao et al., 2019). Consistent with an earlier report on the gut bacterial communities of 58 insect taxa (Colman et al., 2012), these results indicated that the predominant phylum of gut bacteria varies across insect taxa.

### The morphological adaptation of *Diestrammena japanica* to the cave environment

To adapt to the cave environment, cave animals are often small in bodily size. There was a strong positive correlation between oxygen concentration and insect body size. For instance, hypoxic insects exhibited smaller (Harrison et al., 2010). The bodily size of *D. japanica* in the cave regions (weak light and dark regions) was significantly smaller than that in the light regions. In addition, species of *D. japanica* in the cave consume animal feces (Li, 2007), which implies that more pathogens could be introduced by food resources (Busvine, 1980; Hassan et al., 2021). This is consistent with the high abundance of pathogenic Chlamydiae (Candidatus *Rhabdochlamydia porcellionis*) in gut bacteria of *D. japanica* in the dark regions (Busvine, 1980; Hassan et al., 2021), which could damage the digestive system of host insect (Kostanjsek and Pirc Marolt, 2015). Thus, we inferred that the small bodily size of *D. japanica* is relevant to the pathogen presence and lower oxygen concentration inside the cave.

### The contribution of gut bacteria to cave adaptation of *Diestrammena japanica*

Firmicutes were the prominent group of gut bacteria of *D. japanica*, and they may play a significant role in the food digestion and energy metabolism of hosts (Chen et al., 2016), for instance, *Clostridia thermocellum* and *C. ljungdahlii* can degrade the cellulose and hemicellulose (Fonknechten et al., 2010). *Enterococcus* has the ability to mediate the pH of the gut environment, thereby enhancing the host’o resistance to toxins from foods (Wilson and Benoit, 1993; Xia et al., 2018). *Lactobacillus amylovorus* and *L. oligofermentans* were prevalent in our results, with the former exhibiting proteolytic activity and lactose metabolic capacity, producing lactate and carbohydrate assimilation (Mok et al., 2002; Hynönen et al., 2014; Cardarelli et al., 2016), and the latter engaging in the fermentation of xylose, ribose, and hexose (Andreevskaya et al., 2016). Thus, we have reason to believe that a high abundance of Firmicutes in gut bacteria probably supplies *D. japanica* with nutrition substrates *via* involvement in the energy-absorbing processes.

Insects have lost the capacity to independently synthesize essential amino acids throughout their evolutionary history, implying that they rely on gut bacteria or diet to apply these amino acids and nutrients (McCutcheon et al., 2009; Luan et al., 2015). For instance, the artificial diet treatment showed that *Anoplophora glabripennis* (Coleoptera: Cerambycidae) larvae rely on gut bacteria for essential amino acids (Ayayee et al., 2016). Duplais et al. demonstrated that gut bacteria are involved in the cuticular formation of turtle ants Duplais et al. (2021). In this case, the PICRUSt analysis uncovered that gut bacteria of *D. japanica* have the ability to involve in various amino acid biosynthesis and nutritional functions. Generally, compared to herbivorous insects, omnivorous *D. japanica* consume moss, animal carcasses, and fungus (Li, 2007), suggesting that they need a more diverse gut microbiota to decompose foods (Yun et al., 2014; Salcedo-Porras et al., 2020).

Moreover, in gut bacterial communities of species from cave habitats (weak light and dark regions), higher values of connectance and average degree but lower values of modularity represented that each group had more links with other species and fewer subgroups than those in light regions (Wood et al., 2017). This is also supported by the abundance of positive links in the gut bacterial community of *D. japanica*, exhibiting lots of cooperative interactions and lower efficiency of substrate transportation. Gut bacteria with a low abundance of amino acid biosynthesis pathways and more residual amino acids in the ingluvies of *D. japanica* indicated that species in cave habitats tend to have lower metabolism rates in cave habitats than in light regions. To reduce the rate of energy consumption, insects show activity, and mobility tends to be lower in cave environments than outside caves (Howarth and Moldovan, 2018), which is an adaptation of insects to the cave habitats where food and nitrogen are oligotrophic (Barton and Jurado, 2007; White and Culver, 2011).

This research aggregated 15 individual guts from each light region of each cave as a sample, which has the potential to decrease the number of species in the community with skewed compositions and to boost the convergence of patterns in bacterial compositions and network structures. Compared to the metagenomic data of bacterial communities, the 16S rRNA gene data can provide a biased similarity of bacterial communities among samples (Ellegaard et al., 2020). In addition, the identical environment acting as a filter factor can result in a convergent co-occurrence network pattern (Freilich et al., 2018). Thus, microbiome analysis with more replicates would provide more clear patterns for functional predictions of gut bacteria in insect adaptations to cave environments. In addition, more than 40% of gut bacteria may be involved in the nutrient transport and transformation, lipid metabolism, energy metabolism, and amino acid transport processes of *D. japanica*, uncovered by PICRUSt analysis. It is worth noting that these data do not indicate that the gut microbes associated with *D. japanica* do, indeed, perform various important metabolic processes, which would require further experimental studies to be demonstrated. Nevertheless, the characteristics of the gut bacterial community of *D. japanica* revealed in this study provide a basis for exploring the roles of gut bacteria in adapting hosts to caves.

## Data availability statement

The datasets presented in this study can be found in online repositories. The names of the repository/repositories and accession number(s) can be found below: https://doi.org/10.6084/m9.figshare.20522079.v1.

## Author contributions

YD and QC: conceptualization, data curation, and writing the original draft. ZF: visualization and investigation. QWu: formal analysis and validation. LX and XN: data curation and software. QL: methodology and visualization. LT: conceptualization and reviewing. QWe: reviewing, supervising, and funding acquisition. All authors contributed to the article and approved the submitted version.

## Footnotes

1
www.majorbio.com

2
http://cran.r-project.org/

## Supplementary material

Supplementary Table 1 Detail information of sample location.

Supplementary Table 2 Statistical information of 16S rRNA gene deep sequencing.

Supplementary Table 3 OTUs table based on 97% sequence similarity.

Supplementary Table 4 The pairwise Wilcox test of alpha diversity indexes of the gut bacterial community.

Supplementary Table 5 Summary table for the PERMANOVA testing of bacterial compositions in three light-strength regions.
