# Supplementary material for: Gut bacteria reflect the adaptation of Diestrammena japanica (Orthoptera: Rhaphidophoridae) to the cave
Source: Front Microbiol. 2022 Dec 21;13:1016608. doi: 10.3389/fmicb.2022.1016608 (PMC9812492; doi:10.3389/fmicb.2022.1016608)
Supplement: Supplementary Table 1 — Detail information of sample location. [file Table_1.docx]

**Supplementary Table S1** Detail information of sample location

| Caves | North latitude | Eastern longitude | Altitude (m) |
| --- | --- | --- | --- |
| 1 | 25°25′50″ | 107°48′47″ | 770.0 |
| 2 | 25°26′26″ | 107°47′18″ | 850.0 |
| 3 | 25°31′09″ | 107°40′25″ | 836.9 |
