# Supplementary material for: Gut bacteria reflect the adaptation of Diestrammena japanica (Orthoptera: Rhaphidophoridae) to the cave
Source: Front Microbiol. 2022 Dec 21;13:1016608. doi: 10.3389/fmicb.2022.1016608 (PMC9812492; doi:10.3389/fmicb.2022.1016608)
Supplement: Supplementary Table 2 — Statistical information of 16S rRNA gene deep sequencing. [file Table_2.docx]

**Supplementary Table S2** Statistical information of 16S rRNA gene deep sequencing

| Samples | Seq number | Base number | Mean length | Min length | Max length |
| --- | --- | --- | --- | --- | --- |
| L1 | 35686 | 15985190 | 447.9401 | 369 | 483 |
| L2 | 39789 | 17703844 | 444.9432 | 310 | 513 |
| L3 | 34597 | 15359774 | 443.9626 | 271 | 536 |
| WL1 | 51375 | 22880484 | 445.3622 | 266 | 454 |
| WL2 | 36636 | 16386394 | 447.2757 | 269 | 489 |
| WL3 | 84618 | 37427554 | 442.312 | 372 | 487 |
| D1 | 36889 | 16382469 | 444.1017 | 272 | 553 |
| D2 | 36221 | 16269217 | 449.1653 | 310 | 451 |
| D3 | 32208 | 14462166 | 449.024 | 282 | 531 |

L, Light region of cave; WL, Weak light region of cave; D, Darkness region of cave.
