# Supplementary material for: Gut bacteria reflect the adaptation of Diestrammena japanica (Orthoptera: Rhaphidophoridae) to the cave
Source: Front Microbiol. 2022 Dec 21;13:1016608. doi: 10.3389/fmicb.2022.1016608 (PMC9812492; doi:10.3389/fmicb.2022.1016608)
Supplement: Supplementary Table 5 — Summary table for the PERMANOVA testing of bacterial compositions in three light-strength regions. [file Table_5.docx]

**Table S5**. Summary table for the PERMANOVA testing of bacterial compositions in three light-strength regions.

|  | df | Sum of squares | R^2^ | Pseudo-*F* | *P*-value | Permutations |
| --- | --- | --- | --- | --- | --- | --- |
| group | 2 | 0.33709 | 0.31687 | 1.3915 | 0.1288 | 9999 |
| Residual | 6 | 0.72673 | 0.68313 |  |  |  |
| Total | 8 | 1.06382 | 1.00000 |  |  |  |
